# Clinical and mutation profile of pediatric patients with RASopathy-associated hypertrophic cardiomyopathy: results from a Chinese cohort

**DOI:** 10.1186/s13023-019-1010-z

**Published:** 2019-02-07

**Authors:** Hao Chen, Xin Li, Xiaoliang Liu, Jian Wang, Zhen Zhang, Jinjin Wu, Meirong Huang, Ying Guo, Fen Li, Xiumin Wang, Lijun Fu

**Affiliations:** 1 Department of Cardiology, Shanghai Children’s Medical Center, Shanghai Jiao Tong University School of Medicine, Shanghai, 200127 China; 2 Department of Endocrinology and Metabolism, Shanghai Children’s Medical Center, Shanghai Jiaotong University School of Medicine, Shanghai, 200127 China; 3 Department of Pediatric Cardiology, West China Second University Hospital, Sichuan University, Sichuan, 610041 China; 4 Research Division of Birth Defects, Shanghai Children’s Medical Center, Pediatric Translational Medicine Institute, Shanghai Jiaotong University School of Medicine, Shanghai, 200127 China; 5 Shanghai Children’s Medical Center, Shanghai Pediatric Congenital Heart Disease Institute and Pediatric Translational Medicine Institute, Shanghai Jiao Tong University School of Medicine, Shanghai, 200127 China

**Keywords:** RAS-MAPK pathway, Hypertrophic cardiomyopathy, RASopathy

## Abstract

**Background:**

The RASopathies are a class of developmental disorders caused by germline mutations in the RAS-mitogen-activated protein kinase (MAPK) pathway. Hypertrophic cardiomyopathy (HCM) has been frequently described in children with RASopathy, but only a minority of patients have received formal genotyping. The purpose of this study was to evaluate the genetic basis and clinical outcome of pediatric patients with RASopathy-associated HCM.

**Methods:**

We retrospectively reviewed the mutation spectrum and clinical outcome of all the patients with RASopathy derived from 168 pediatric HCM cases referred to our institution between January 2012 and July 2018.

**Results:**

A heterozygous missense mutation in one of known RASopathy genes was identified in 46 unrelated children with HCM. Mutations in the *PTPN11* gene were the most prevalent (19/46); this was followed by mutations in *RAF1* (11/46), *KRAS* (5/46), *RIT1* (4/46), *BRAF* (3/46), *SOS1* (2/46), *HRAS* (1/46), and *SHOC2* (1/46). Moreover, two compound heterozygous missense mutations in the *LZTR1* gene were identified in one patient with the Noonan syndrome phenotype and HCM. The median age at the diagnosis of HCM was 3.0 months (range 0 months to 8.1 years). Twenty-one of the patients had significant left ventricular outflow tract obstruction and 32 had concomitant congenital heart disease. Three patients with a mutation in exon 13 of the *PTPN11* gene died of cardiac failure at the ages of 3.0, 3.5, and 6.0 months. The remaining 44 patients were alive after an average follow-up time of 3.9 years (0.5 to 17.1 years, median 2.9 years) from the initial diagnosis of HCM, including 5 patients with spontaneous regression of their cardiac hypertrophy.

**Conclusions:**

RASopathy-associated HCM is a heterogeneous genetic condition characterized by early-onset cardiac hypertrophy and a high prevalence of co-existing congenital heart disease, which is most frequently related to specific mutations in the *PTPN11* gene. Rapidly progressive HCM, resulting in an early death, is uncommon in RASopathy patients except those with specific mutations in exon 13 of the *PTPN11* gene.

## Background

Noonan syndrome (NS) is a relatively common genetic condition with an incidence of 1 in every 1000–2500 live births [1]. Hypertrophic cardiomyopathy (HCM) is present in approximately 20 to 30% of individuals with NS and represents a major determinant of outcome in these patients. Several genetic disorders phenotypically related to NS are also frequently associated with HCM, such as Noonan syndrome with multiple lentigines (NSML), cardiofaciocutaneous syndrome (CFCS), Costello syndrome (CS), and Noonan syndrome-like disorder with loose anagen hair (NS/LAH) [2].

NS and related syndromes, also known as the RASopathies, are caused by germline mutations in the RAS-mitogen-activated protein kinase (MAPK) pathway. More than twenty genes have been reported to be associated with RASopathies, including *A2ML1, BRAF, CBL, HRAS, KRAS, LZTR1, MAP2K1, MAP2K2, NF1, NRAS, PPP1CB, PTPN11, RAF1, RASA1, RASA2, RIT1, RRAS, SHOC2, SOS1, SOS2,* and *SPRED1* [3]. Owing to their similar pathogenetic mechanisms, these syndromes share many clinical features such as distinct facial features, congenital heart defects, cardiomyopathy, skin anomalies, and growth retardation. In the majority of cases, the diagnosis of RASopathy can be made clinically by the identification of the typical clinical features. However, the syndromic manifestations can be subtle or even absent for some patients, making RASopathy-associated HCM difficult to distinguish from non-syndromic HCM on clinical grounds alone.

HCM has been frequently described in children with RASopathy, but only a minority of study patients have received formal genotyping in previous studies [4, 5]. The genetic basis and genotype-phenotype correlation of pediatric HCM associated with RASopathy still remain to be elucidated. In the current study, we present the mutation spectrum and clinical outcome of 47 unrelated children with RASopathy and HCM, which demonstrated that NSML-associated *PTPN11* mutation was the most common cause of RASopathy-associated HCM. We also found that rapidly progressive HCM was uncommon in RASopathy patients, except those with specific mutations in exon 13 of the *PTPN11* gene.

## Methods

### Patients and clinical evaluation

The study cohort comprised all the patients with RASopathy derived from 168 pediatric HCM cases referred to our institution between January 2012 and July 2018. Children with cardiac hypertrophy secondary to inborn errors of metabolism were excluded. HCM was defined as left ventricular posterior and/or septal wall thickness > 2 standard deviations above the normal mean for body surface area in the absence of hemodynamic stresses sufficient to account for the degree of hypertrophy [6]. A left ventricular (LV) outflow tract gradient ≥30 mmHg was considered to represent significant left ventricular outflow tract obstruction (LVOTO). All patients were clinically assessed by experienced clinical geneticists and pediatric cardiologists. Cardiovascular evaluation was performed on all the patients, including baseline electrocardiogram and echocardiography at the time of clinical diagnosis and during follow-up.

### Molecular genetic analysis

Peripheral blood was collected, and genomic DNA was extracted according to standard procedures. Genetic testing was performed by next-generation sequencing (NGS) using either an expanded cardiomyopathy panel (58 genes, including 12 known RASopathy genes: *BRAF, CBL, HRAS, KRAS, MAP2K1, MAP2K2, NF1, NRAS, PTPN11, RAF1, SHOC2,* and *SOS1*) or a whole-exome sequencing approach (Illumina HiSeq2500). Alignment of sequence reads to a reference human genome (Human 37.3, SNP135) was performed using the NextGENe® software (SoftGenetics, State College, Pennsylvania, USA). All single nucleotide variants and indels were saved as VCF format files, and uploaded to Ingenuity® Variant Analysis™ (Ingenuity Systems, Redwood City, California, USA) for variation filtering and interpretation. All the variations were classified according to the recommended method of the American College of Medical Genetics and Genomics [7]. Pathogenic and potentially pathogenic mutations were confirmed by Sanger sequencing, where possible, and validated by parental testing and segregation analysis. Variants classified as likely benign or benign were not selected to validate using Sanger sequencing.

### Analysis of the pathogenicity of novel missense variants

To determine the potential pathogenicity of novel missense variants, we used three in silico prediction methods: MutationTaster (http://www.mutationtaster.org), Sorting Tolerant from Intolerant (SIFT, http://sift.jcvi.org/), and Polymorphism Phenotyping v2 (PolyPhen-2, http://genetics.bwh.harvard.edu/pph2/).

### Treatment and follow-up

β-Blocker therapy had been prescribed in 14 subjects with LVOTO. Balloon pulmonary valvuloplasty for treatment of pulmonary valve stenosis (PVS) was undertaken in one patient (patient 20 in Table 1). Six patients (patients 09, 23, 24, 37, 38, and 45 in Table 1) received surgical therapy for concomitant congenital heart disease (CHD), such as atrial septal defect (ASD), PVS, ventricular septal defect, and patent ductal arteriosus. Three patients (patient 18, 21, and 29 in Table 1) received surgical myectomy as a treatment for HCM. All patients were followed up by either telephone interview or outpatient clinic visit.

**Table 1:** The cardiac manifestations and mutation profiles of 47 pediatric patients with RASopathy and HCM

Case	Sex	Age HCM Diagnosed (months)	Clinical phenotype	LVOTO	Associated cardiac defect	Gene Mutation	Origin of mutation	ACMG classification	Follow-up time (years)
01	M	1.7	NSML	+	ASD	*PTPN11*, c.836A > G (p.Y279C)	De novo	Pathogenic	7.1
02	M	12	NSML	+	–	*PTPN11*, c.836A > G (p.Y279C)	De novo	Pathogenic	15.2
03	M	1.0	NSML	–	PVS	*PTPN11*, c.836A > G (p.Y279C)	De novo	Pathogenic	1.4
04	F	2 d	NS	+	–	*PTPN11*, c.836A > G (p.Y279C)	De novo	Pathogenic	1.2
05	M	6.0	NS	–	–	*PTPN11*, c.836A > G (p.Y279C)	De novo	Pathogenic	0.5
06	F	10	NS	–	ASD, PVS	*PTPN11*, c.854 T > C (p.F285S)	De novo	Pathogenic	1.6
07	M	3 d	–	–	ASD	*PTPN11*, c.1391G > C (p.G464A)	De novo	Pathogenic	2.0
08	M	10	NS	+	–	*PTPN11*, c.1492C > T (p.R498W)	Mother	Pathogenic	6.8
09	M	Fetal	NS	+	ASD, PVS	*PTPN11*, c.1517A > C (p.Q506P)	De novo	Pathogenic	4.0
10	F	8 d	NS	–	ASD, PDA	*PTPN11*, c.1517A > C (p.Q506P)	De novo	Pathogenic	1.6
11	M	17 d	NS	–	ASD, PVS	*PTPN11*, c.1517A > C (p.Q506P)	De novo	Pathogenic	Die at 6.0 ms old
12	F	Fetal	NS	+	–	*PTPN11*, c.1528C > G (p.Q510E)	De novo	Pathogenic	4.9
13	F	2.5	–	+	ASD	*PTPN11*, c.1528C > G (p.Q510E)	De novo	Pathogenic	Die at 3.0 ms old
14	M	1.0	NS	+	–	*PTPN11*, c.1528C > G (p.Q510E)	De novo	Pathogenic	8.3
15	F	1.5	NS	+	PVS	*PTPN11*, c.1528C > G (p.Q510E)	De novo	Pathogenic	3.9
16	M	1 d	NS	+	VSD	*PTPN11*, c.1528C > G (p.Q510E)	De novo	Pathogenic	0.7
17	M	1 d	NS	+	VSD	*PTPN11*, c.1528C > G (p.Q510E)	De novo	Pathogenic	Die at 3.5 ms old
18	M	3 d	NS	+	–	*PTPN11*, c.1528C > G (p.Q510E)	De novo	Pathogenic	2.0
19	F	1.3	NS	+	–	*PTPN11*, c.1528C > G (p.Q510E)	De novo	Pathogenic	9.3
20	F	9.0	NS	+	ASD, PVS	*RAF1*, c.770C > T (p.S257 L)	De novo	Pathogenic	3.9
21	F	8.0	NSML	+	–	*RAF1*, c.770C > T (p.S257 L)	De novo	Pathogenic	13.1
22	M	12	NS	–	VSD, ASD	*RAF1*, c.770C > T (p.S257 L)	De novo	Pathogenic	1.8
23	M	2 d	NS	–	ASD	*RAF1*, c.770C > T (p.S257 L)	De novo	Pathogenic	1.1
24	F	30	NS	–	ASD	*RAF1*, c.770C > T (p.S257 L)	De novo	Pathogenic	6.5
25	M	6.0	NS	+	ASD, PVS	*RAF1*, c.770C > T (p.S257 L)	De novo	Pathogenic	1.2
26	F	4.0	NS	–	DORV, VSD, ASD, PVS	*RAF1*, c.781C > A (p.P261T)	De novo	Pathogenic	3.6
27	F	5.5	NS	–	–	***RAF1*** **c.786 T > G (p.N262K)** ^**a**^	De novo	Pathogenic	7.4
28	F	12	NS	+	ASD	***RAF1*** **, c.788 T > G (p.V263G)** ^**a**^	De novo	Pathogenic	1.1
29	M	17	NS	+	–	*RAF1,* c.1472C > T (p.T491I)	NA	Pathogenic	1.3
30	M	8.1y	NS	–	ASD	*RAF1*,c.1837C > G (p.L613 V)	De novo	Pathogenic	3.7
31	F	16 d	NS	–	ASD, PVS	*KRAS*, c.101C > T (p.P34L)	De novo	Pathogenic	0.9
32	M	34	NS	–	ASD, LSVC	*KRAS*, c.178G > A (p.G60S)	De novo	Pathogenic	2.0
33	M	2.0	NS	–	–	*KRAS*, c.458A > T (p.D153V)	De novo	Pathogenic	3.0
34	M	15 d	NS	–	ASD, PDA	*KRAS*, c.458A > T (p.D153V)	De novo	Pathogenic	0.9
35	F	16	NS	–	ASD, PVS	*KRAS*, c.458A > T (p.D153V)	De novo	Pathogenic	0.8
36	F	3.0	NS	–	ASD, PVS	*RIT1*, c.170C > G (p.A57G)	De novo	Pathogenic	0.9
37	F	17	NS	–	ASD, PVS, PDA	*RIT1*, c.170C > G (p.A57G)	De novo	Pathogenic	1.4
38	F	6.8	NS	–	ASD, PVS,VSD	*RIT1*, c.170C > G (p.A57G)	De novo	Pathogenic	4.0
39	M	2.4	NS	–	ASD, PVS	*RIT1*, c.270G > C (p.M90I)	De novo	Pathogenic	4.2
40	M	3.5	CFCS	–	–	*BRAF*, c.770A > G (p.Q257R)	De novo	Pathogenic	0.9
41	F	5.0	CFCS	–	ASD, PVS	*BRAF*, c.1502A > G (p.E501G)	De novo	Pathogenic	0.6
42	F	5.0	CFCS	–	VSD	***BRAF*** **, c.1796C > T (p.T599I)** ^**a**^	De novo	Pathogenic	3.7
43	M	6.0y	NS	+	–	*SOS1* c.1644 T > G (p.S548R)	De novo	Pathogenic	7.4
44	M	3.0	NS	+	ASD	*SOS1*, c.2536G > A (p.E846K)	De novo	Pathogenic	17.1
45	M	3.0	CS	–	ASD, PVS	*HRAS*, c.179G > A (p.G60D)	De novo	Pathogenic	3.8
46	M	3.0	NS/LAH	–	–	*SHOC2*, c.4A > G (p.S2G)	De novo	Pathogenic	2.7
47	M	1.0	NS	+	ASD	***LZTR1*** **, c.509G > C(p.R170P)** ^**a**^ ***LZTR1*** **, c.2374 T > G(p.C792G)** ^**a**^	Mother Father	Likely pathogenic Likely pathogenic	4.2

*HCM* hypertrophic cardiomyopathy, *LVOTO* left ventricular outflow tract obstruction, *ACMG* American College of Medical Genetics and Genomics, *NSML* Noonan syndrome with multiple lentigines, *ASD* atrial septal defect, *PVS* pulmonary valve stenosis, *NS* Noonan syndrome, *PDA* patent duct arteriosus, *DORV* double outlet right ventricle, *VSD* ventricular septal defect, *NA* not available, *LSVC* left superior vena cava, *CFCS* cardiofaciocutaneous syndrome, * CS* Costello syndrome, *NS/LAH* Noonan syndrome with loose anagen hair

^a^The mutation identified in this study is in boldface

## Results

### Mutation analysis and clinical phenotype

Germline mutations in one of known RASopathy genes were identified in 47 unrelated children (27 males, 20 females), including 36 patients with the NS phenotype, four with NSML, three with CFCS, one with CS, one with NS/LAH, and two infants without typical syndromic features (e.g., short stature, typical facial dysmorphia, and developmental delay) at the time of inclusion. The majority of patients (30/47, 64%) were diagnosed with HCM before the age of 6 months, including two with prenatal onset and 10 with neonatal onset. The median age at the diagnosis of HCM was 3.0 months (range 0 month to 8.1 years). Of the 47 patients, 21 (45%) had significant LVOTO at initial diagnosis of HCM. Concomitant CHD was observed in 32 of 47 (68%) patients, frequently in the form of septal defects and PVS. The clinical phenotypes and genetic features pertinent to the index cases are described below and summarized in Table 1.

Six different known mutations in the *PTPN11* gene were identified in this series. Mutations in exon 13 were the most common variants, and were found in 12 individuals with early-onset biventricular hypertrophy (BVH). The recurrent mutation p.Q510E was identified in eight patients with biventricular outflow tract obstruction. Seven of these patients presented with the NS phenotype, whereas the other one did not have overt clinical syndromic features at the time of inclusion. Meanwhile, two other missense mutations in exon 13 (p.R498W and p.Q506P) were detected in four individuals with the NS phenotype, including two patients with significant LVOTO. Moreover, one recurrent mutation in exon 7 (p.Y279C) was found in three patients with NSML and two with the NS phenotype. Three of these five patients also had LVOTO. One mutation in exon 12 (p.G464A) was identified in an infant with BVH but without overt clinical syndromic features at the time of inclusion. One mutation in exon 8 (p.F285S) was detected in another infant with the NS phenotype and focal septal hypertrophy.

Six different variants in the *RAF1* gene were identified in this series, which included four in the conserved region two (CR2) domain, one in the activation segment of the kinase domain, and one at the C-terminus of RAF1. The recurrent mutation p.S257 L was found in one girl with NSML and five children with the NS phenotype. Another three known mutations (p.P261T, p.T491I, and p.L613 V) in the *RAF1* gene were detected in three subjects with the NS phenotype. Moreover, two novel variants (p.N262K and p.V263G) were identified in two patients with typical facial appearance of NS, short stature, and severe HCM. Of the 11 patients harboring the *RAF1* mutation, five had significant LVOTO.

Three known mutations (p.P34L, p.G60S, and p.D153V) in the *KRAS* gene were identified in five individuals with the NS phenotype and mild cardiac hypertrophy. Moreover, two previously reported mutations (p.A57G and p.M90I) in the *RIT1* gene were identified in four patients with NS and BVH, all of whom had co-existing PVS and ASD. Additionally, two known mutations (p.Q257R and p.E501G) in the *BRAF* gene were identified in two individuals with CFCS and BVH. One novel variant (p.T599I) in the *BRAF* gene was identified in a girl with macrocephaly, hypertelorism, dark-colored moles, developmental delays, intellectual disabilities, ventricular septal defect, and focal septal hypertrophy. Furthermore, two known mutations (p.S548R and p.E846K) in the *SOS1* gene were identified in two individuals with NS phenotypes and LVOTO.

In two other genes, a previously reported missense mutation was found in a single individual: one mutation (p.G60D) in *HRAS* was detected in a patient with CS and severe LV hypertrophy; and one mutation (p.S2G) in *SHOC2* was detected in a patient with NS/LAH and moderate LV hypertrophy.

Moreover, two novel compound heterogeneous variants in the *LZTR1* gene (p.R170P and p.C792G) were identified in one patient with the typical facial appearance of NS, short stature, and severe LV hypertrophy. Family screening for the variants revealed that each of his parents was the carrier of one of these variants, his brother was heterozygote carrier for the p.R170P variant, and his maternal half-sister was wild-type for both variants. Neither his parents nor his siblings were clinically affected.

### Analysis of the pathogenicity of novel missense variants

Five novel variants were identified in this study, including two de novo variants (p.N262K and p.V263G) in the *RAF1* gene, one de novo variant (p.T599I) in the *BRAF* gene, and two compound heterogeneous variants (p.R170P and p.C792G) in the *LZTR1* gene*.* All affected residues were evolutionarily conserved, and no change had been reported in Genome Aggregation Database (gnomAD, http://gnomad.broadinstitute.org). Moreover, all five variants were consistently predicted to be deleterious by three bioinformatics tools (Table 2).

**Table 2:** In silico evaluation of five novel mutations

Gene	DNA change	Protein change	Mutation taster	PolyPhen-2	SIFT	MAF (gnomAD)
*RAF1*	c.786 T > G	p.N262K	Disease-causing	Probably damaging	Deleterious	–
*RAF1*	c.788 T > G	p.V263G	Disease-causing	Probably damaging	Deleterious	–
*BRAF*	c.1796C > T	p.T599I	Disease-causing	Probably damaging	Deleterious	–
*LZTR1*	c.509G > C	p.R170P	Disease-causing	Probably damaging	Deleterious	–
*LZTR1*	c.2374 T > G	p.C792G	Disease-causing	Probably damaging	Deleterious	–

### Follow-up and survival

Three patients with a mutation in exon 13 of the *PTPN11* gene had rapidly progressive HCM and died of cardiac failure at the age of 3.0, 3.5, and 6.0 months, respectively. The remaining 44 patients were alive and received an average follow-up time of 3.9 years (0.5 to 17.1 years, median 2.9 years) from the initial diagnosis of HCM. As assessed by serial echocardiographic follow-up, five patients had spontaneous regression of ventricular wall thickness over an average of 5.7 years (0.5 to 15.4 years, median 5.7 years), including two with mutations in *PTPN11*, two with mutations in *KRAS*, and one with a mutation in *SOS1*. Progression of HCM was seen in 4 patients with mutations in *PTPN11*, one with a mutation in *SOS1,* and one with mutations in *LZTR1*. HCM was stabilized in the remaining 33 patients, including three patients who have received surgical myectomy for HCM.

## Discussion

Germline *PTPN11* mutations are major causes for both NS and NSML, but the point mutations are almost mutually exclusive between these two conditions [8]. Of the six different *PTPN11* mutations identified in the study, only the p.F285S variant has been regarded as an NS-associated *PTPN11* mutation, while the other five mutations appeared to be specific for NSML according to previous studies [9]. However, among the 18 patients with an NSML-associated *PTPN11* mutation, only three patients presented with the NSML phenotype at the time of inclusion, while the other 15 patients did not fulfill the criteria for a clinical diagnosis of NSML according to Voron et al. [10]. These findings indicated that NSML-associated *PTPN11* mutations were the most common causes of HCM associated with RASopathy, whereas the differential diagnosis between NS and NSML may be difficult on clinical grounds alone due to their overlapping clinical features. Our cohort also included two infants without overt clinical syndromic features at the time of inclusion, who had the diagnosis of RASopathy after genetic tests. It is possible that the typical syndromic features may be subtle or even absent in these individuals due to their young age, leading to a clinical diagnosis of isolated HCM [11]. These findings suggested that the inclusion of RASopathy genes in cardiomyopathy genetic testing panels may contribute to the early diagnosis of RASopathy-associated HCM.

HCM is the prevailing cardiac defect in patients with germline *RAF1* mutations [12]. However, only the kinase-activating *RAF1* mutations, which cluster in the CR2 domain or the C-terminus of RAF1, are highly associated with HCM [13]. Surprisingly, one kinase-impaired *RAF1* mutation (p.T491I), which is rarely associated with HCM, was identified in one of our patients with a typical NS phenotype and HCM [13]. Of the six different *RAF1* mutations identified in this study, two are reported here for the first time. The novel variant p.N262K was caused by a c.786 T > G substitution; a different base substitution (c.786 T > A) resulting in the same amino acid substitution has been described in a patient with a CS phenotype [14]. The novel variant p.V263G was caused by a c.788 T > G substitution; a different amino acid change at the same position (p.V263A) has been described in a patient with NS and HCM [15]. Both of the novel variants affected a highly conserved residue within the CR2 domain and were identified as occurring de novo. Both of them were absent in the SNP database and were consistently predicted to be deleterious by three bioinformatics tools. These findings, along with the typical clinical features in the affected patients, indicate that these two variants are pathogenic.

Patients harboring *RIT1* mutations display a high incidence of HCM, similar to those with *RAF1* mutations [16]. PVS and ASD are also prevalent among patients harboring *RIT1* mutations according to previous studies [16]. We identified two different *RIT1* mutations in four patients with NS and HCM, all of whom had co-existing PVS and ASD. Our findings, along with previous studies, indicated that the combination of PVS and ASD with HCM seemed to be frequent among individuals with NS due to *RIT1* mutations [2, 16].

HCM is also a frequent cardiovascular manifestation in patients with CFCS, which was documented in our three patients with CFCS due to a *BRAF* mutation. The novel variant p.T599I affected a highly conserved residue within the activation segment of the CR3 domain, and was identified as occurring de novo. This variant was absent in the SNP database and was consistently predicted to be deleterious by three bioinformatics tools. The same mutation has been reported previously as a somatic mutation in association with several types of cancer according to the Catalogue of Somatic Mutations in Cancer database (https://cancer.sanger.ac.uk/cosmic). Moreover, a different mutation at this residue (p.T599R) has been described in a patient with CFCS [17]. This, along with the typical clinical features in the affected patient, indicates that this variant is pathogenic.

Germline *KRAS* mutations are rarely associated with HCM according to previous studies [18]. In contrast, *KRAS* mutations seemed not to be rare in our cohort. However, only mild HCM was observed in the five patients harboring a *KRAS* mutation. The detailed mechanisms are unclear, but may be attributed to the fact that germline *KRAS* mutations cause only a moderate up-regulation of the RAS-MAPK pathway [19]. Germline *SOS1* mutations are the second most common causes of NS, but the risk of HCM in individuals with *SOS1* mutations is low [20, 21]. Only two patients in our study population were found to harbor a mutation in the *SOS1* gene. HCM is frequently seen in patients with CS caused by *HRAS* mutations and NS/LAH caused by *SHOC2* mutations. However, both of these diseases are rare syndromes within the RASopathy group [2, 22]. Only one patient with an *HRAS* mutation and another patient with a *SHOC2* mutation were found in our study.

Germline *LZTR1* mutations have been shown to be associated with NS in recent studies, which can follow either recessive or dominant inheritance patterns [23, 24]. While a limited number of patients with germline *LZTR1* mutations have been described, it appears that a high percentage of HCM is associated with this condition [24, 25]. In the current study, two novel compound heterogeneous variants in the *LZTR1* gene (p.R170P and p.C792G) were identified in one patient with a typical NS phenotype and HCM. Both of the variants were absent in the SNP database and were consistently predicted to be deleterious by three bioinformatics tools. Moreover, they were found to co-segregate with the disease phenotype in an autosomal recessive fashion in the affected family. This implies that the two variants may be the pathogenic mutations responsible for the NS phenotype in this patient.

An early onset of cardiac hypertrophy was observed in our study population, with a median age of 3.0 months at the diagnosis of HCM. Meanwhile, LVOTO was detected at a high frequency in our cohort, especially in patients with NSML-associated *PTPN11* mutations (13/18, 72%). Furthermore, a high prevalence of co-existing CHD was observed in our study population. These results are consistent with several previous reports [26, 27].

The clinical outcomes of our patients were markedly variable. The disease can be rapidly progressive in infancy in some patients, or remain asymptomatic, or regress in other patients. The varied severity of HCM and clinical outcome may be related to the different gene mutations within the RAS-MAPK pathway. We observed that specific mutations in exon 13 of the *PTPN11* gene were associated with early-onset cardiac hypertrophy and certain mutations such as the p.Q510E variant were associated with rapidly progressive HCM, in agreement with other studies [28, 29]. In contrast, other gene mutations within the RAS-MAPK pathway seemed to be associated with a more benign course. Particularly, spontaneous regression of HCM was observed in five patients in our cohort, but the detailed mechanisms remain to be elucidated.

There are some limitations of our study. Several recently discovered genes, e.g. *A2ML1, LZTR1, RIT1, RRAS, RASA1, RASA2, SOS2*, and *SPRED1* have not been tested in some of the patients in our study. Our RASopathy subjects are relatively young, and the follow-up period is short. Given that a high number of genes may be associated with RASopathies but a relatively small number of RASopathy patients was included in this cohort, our study does not allow any genotype-phenotype correlation analysis in some rare genes. Further investigations into the correlation of specific genetic mutations and clinical outcomes of patients with RASopathy-associated HCM are warranted.

## Conclusions

RASopathies are one of the most common causes of pediatric HCM, characterized by early-onset cardiac hypertrophy as well as a higher prevalence of CHD and LVOTO. RASopathy-associated HCM is a genetically heterogeneous condition involving diverse genes within the RAS-MAPK pathway, but frequently related to NSML-associated *PTPN11* mutations. The clinical course and prognosis of patients with this condition are markedly variable and seemed to be related to specific gene mutations. However, rapidly progressive HCM, resulting in an early death, was uncommon in RASopathy patients except those with specific mutations in exon 13 of the *PTPN11* gene.

## Abbreviations

ASD: Atrial septal defect
BVH: Biventricular hypertrophy
CFCS: Cardiofaciocutaneous syndrome
CHD: Congenital heart disease
CR: Conserved region
CS: Costello syndrome
gnomAD: Genome Aggregation Database
HCM: Hypertrophic cardiomyopathy
LV: Left ventricular
LVOTO: Left ventricular outflow tract obstruction
MAPK: Mitogen-activated protein kinase
NGS: Next-generation sequencing
NS: Noonan syndrome
NS/LAH: Noonan syndrome-like disorder with loose anagen hair
NSML: Noonan syndrome with multiple lentigines
PTP: Protein tyrosine phosphatase
PVS: Pulmonary valve stenosis

## References

[1] Roberts AE, Allanson JE, Tartaglia M, Gelb BD (2013). Noonan syndrome. Lancet.

[2] Gelb BD, Roberts AE, Tartaglia M (2015). Cardiomyopathies in Noonan syndrome and the other RASopathies. Prog Pediatr Cardiol.

[3] Aoki Y, Niihori T, Inoue S, Matsubara Y (2016). Recent advances in RASopathies. J Hum Genet.

[4] Cox GF, Sleeper LA, Lowe AM, Towbin JA, Colan SD, Orav EJ (2006). Factors associated with establishing a causal diagnosis for children with cardiomyopathy. Pediatrics.

[5] Nugent AW, Daubeney PE, Chondros P, Carlin JB, Colan SD, Cheung M (2005). Clinical features and outcomes of childhood hypertrophic cardiomyopathy: results from a national population-based study. Circulation.

[6] Grenier MA, Osganian SK, Cox GF, Towbin JA, Colan SD, Lurie PR (2000). Design and implementation of the North American Pediatric Cardiomyopathy Registry. Am Heart J.

[7] Richards S, Aziz N, Bale S, Bick D, Das S, Gastier-Foster J (2015). Standards and guidelines for the interpretation of sequence variants: a joint consensus recommendation of the American College of Medical Genetics and Genomics and the Association for Molecular Pathology. Genet Med.

[8] Kontaridis MI, Swanson KD, David FS, Barford D, Neel BG (2006). PTPN11 (Shp2) mutations in LEOPARD syndrome have dominant negative, not activating, effects. J Biol Chem.

[9] Sarkozy A, Digilio MC, Dallapiccola B (2008). Leopard syndrome. Orphanet J Rare Dis.

[10] Voron DA, Hatfield HH, Kalkhoff RK (1976). Multiple lentigines syndrome. Case report and review of the literature. Am J Med.

[11] Ceyhan-Birsoy O, Miatkowski MM, Hynes E, Funke BH, Mason-Suares H (2018). NGS testing for cardiomyopathy: utility of adding RASopathy-associated genes. Hum Mutat.

[12] Tartaglia M, Gelb BD (2010). Disorders of dysregulated signal traffic through the RAS-MAPK pathway: phenotypic spectrum and molecular mechanisms. Ann N Y Acad Sci.

[13] Pandit B, Sarkozy A, Pennacchio LA, Carta C, Oishi K, Martinelli S (2007). Gain-of-function RAF1 mutations cause Noonan and LEOPARD syndromes with hypertrophic cardiomyopathy. Nat Genet.

[14] Kobayashi T, Aoki Y, Niihori T, Cave H, Verloes A, Okamoto N (2010). Molecular and clinical analysis of RAF1 in Noonan syndrome and related disorders: dephosphorylation of serine 259 as the essential mechanism for mutant activation. Hum Mutat.

[15] Razzaque MA, Nishizawa T, Komoike Y, Yagi H, Furutani M, Amo R (2007). Germline gain-of-function mutations in RAF1 cause Noonan syndrome. Nat Genet.

[16] Yaoita M, Niihori T, Mizuno S, Okamoto N, Hayashi S, Watanabe A (2016). Spectrum of mutations and genotype-phenotype analysis in Noonan syndrome patients with RIT1 mutations. Hum Genet.

[17] Sarkozy A, Carta C, Moretti S, Zampino G, Digilio MC, Pantaleoni F (2009). Germline BRAF mutations in Noonan, LEOPARD, and cardiofaciocutaneous syndromes: molecular diversity and associated phenotypic spectrum. Hum Mutat.

[18] Zenker M, Lehmann K, Schulz AL, Barth H, Hansmann D, Koenig R (2007). Expansion of the genotypic and phenotypic spectrum in patients with KRAS germline mutations. J Med Genet.

[19] Dalin MG, Zou Z, Scharin-Tang M, Safari R, Karlsson C, Bergo MO (2014). Myocardial KRAS(G12D) expression does not cause cardiomyopathy in mice. Cardiovasc Res.

[20] Rauen KA (2013). The RASopathies. Annu Rev Genomics Hum Genet.

[21] Lepri F, De Luca A, Stella L, Rossi C, Baldassarre G, Pantaleoni F (2011). SOS1 mutations in Noonan syndrome: molecular spectrum, structural insights on pathogenic effects, and genotype-phenotype correlations. Hum Mutat.

[22] Gripp KW, Lin AE (2012). Costello syndrome: a Ras/mitogen activated protein kinase pathway syndrome (rasopathy) resulting from HRAS germline mutations. Genet Med..

[23] Yamamoto GL, Aguena M, Gos M, Hung C, Pilch J, Fahiminiya S (2015). Rare variants in SOS2 and LZTR1 are associated with Noonan syndrome. J Med Genet.

[24] Johnston JJ, van der Smagt JJ, Rosenfeld JA, Pagnamenta AT, Alswaid A, Baker EH, et al. Autosomal recessive Noonan syndrome associated with biallelic LZTR1 variants. Genet Med. 2018. 10.1038/gim.2017.249. 10.1038/gim.2017.249 PMC6105555 29469822

[25] Umeki I, Niihori T, Abe T, Kanno SI, Okamoto N, Mizuno S, et al. Delineation of LZTR1 mutation-positive patients with Noonan syndrome and identification of LZTR1 binding to RAF1-PPP1CB complexes. Hum Genet. 2018. 10.1007/s00439-018-1951-7. 10.1007/s00439-018-1951-7 30368668

[26] Hickey EJ, Mehta R, Elmi M, Asoh K, McCrindle BW, Williams WG (2011). Survival implications: hypertrophic cardiomyopathy in Noonan syndrome. Congenit Heart Dis.

[27] Wilkinson JD, Lowe AM, Salbert BA, Sleeper LA, Colan SD, Cox GF (2012). Outcomes in children with Noonan syndrome and hypertrophic cardiomyopathy: a study from the pediatric cardiomyopathy registry. Am Heart J.

[28] Hahn A, Lauriol J, Thul J, Behnke-Hall K, Logeswaran T, Schanzer A (2015). Rapidly progressive hypertrophic cardiomyopathy in an infant with Noonan syndrome with multiple lentigines: palliative treatment with a rapamycin analog. Am J Med Genet A.

[29] Takahashi K, Kogaki S, Kurotobi S, Nasuno S, Ohta M, Okabe H (2005). A novel mutation in the PTPN11 gene in a patient with Noonan syndrome and rapidly progressive hypertrophic cardiomyopathy. Eur J Pediatr.

